# Arthroscopic Excision of Intra-Articular Osteoid Osteoma at the Elbow

**DOI:** 10.1155/2019/8505382

**Published:** 2019-02-25

**Authors:** Taku Hatta, Masami Hosaka, Munenori Watanuki, Toshihisa Yano, Shinichirou Yoshida, Mika Watanabe, Shin Hitachi, Eiji Itoi

**Affiliations:** ^1^Department of Orthopaedic Surgery, Tohoku University School of Medicine, Japan; ^2^Department of Pathology, Tohoku University School of Medicine, Japan; ^3^Department of Diagnostic Radiology, Tohoku University School of Medicine, Japan

## Abstract

Osteoid osteoma (OO) apparent in the intra-articular region of the elbow is very rare. Although computed tomography-guided excision and radiofrequency ablation have been recognized as useful treatment options, arthroscopic excision has recently received focus as an alternative strategy for lesions close to neurovascular structures or intra- and juxta-articular lesions. We herein report a 17-year-old female who underwent arthroscopic treatment for intra-articular OO located at the olecranon/coronoid fossa. Her symptoms included elbow pain that was exacerbated at night and contracture of elbow flexion-extension, and she was diagnosed with intra-articular OO after 12 months of symptomatic history. Arthroscopically, thorough synovectomy for both the anterior and posterior aspects of the joint enabled definition of the tumor margin with hyperemic alteration and excision of the lesion as an en bloc specimen. At the 12-month follow-up, the patient had no recurrence of elbow limitation or pain. This case report describes the advantages of arthroscopic treatment, including a low-invasive approach and easy accessibility to the whole intra-articular space, which can provide clear visualization of the tumorous lesion.

## 1. Introduction

Osteoid osteoma (OO) is a benign bone tumor generally apparent in the first to third decades. It has been reported that approximately 50% of OOs occur in the femur and tibia with the diaphyseal shaft or toward the metaphysis, with the elbow joint very rarely affected [1]. Although recognizing the characteristic symptoms, which include regional pain that is increasingly severe at night and significantly relieved by medication with nonsteroidal anti-inflammatory drugs, can aid in the diagnosis of OO, its accurate diagnosis remains challenging, especially in cases of intra- or juxta-articular OOs, since more common disorders, such as monoarticular arthritis and tendinitis, easily mimic the clinical features of OOs [2, 3].

In addition to tumor excision through the open approach, computed tomography- (CT-) guided excision and radiofrequency ablation have been recognized as viable treatment options [4, 5]. Arthroscopic excision has recently received focus as an alternative, especially for lesions close to the neurovascular structures or intra- and juxta-articular lesions [6]. We herein report a patient with intra-articular OO at the elbow that was successfully treated with an arthroscopic procedure. The patient and her family were informed that the data would be submitted for publication and gave their consent.

## 2. Case Presentation

**Figure 1:**
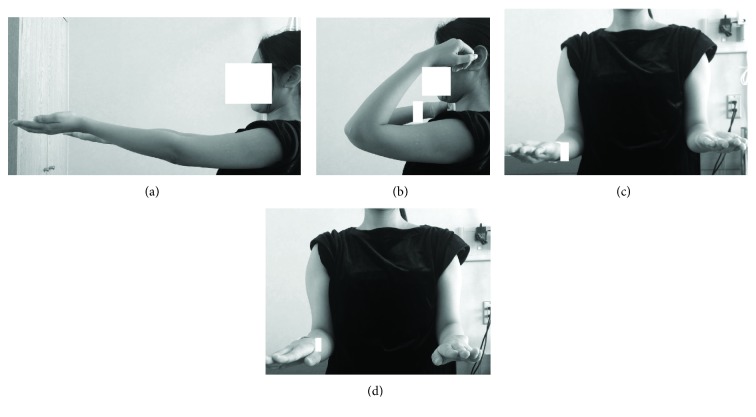
Preoperative ranges of motion. Elbow extension (a) and flexion (b). Forearm supination (c) and pronation (d).

**Figure 2:**
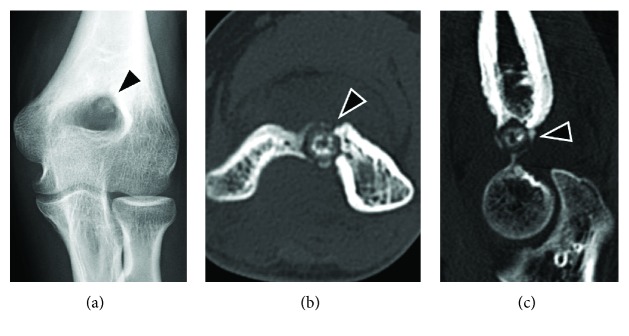
Preoperative radiographic images. The anteroposterior radiograph (a) and computed tomography images (b: axial, c: sagittal) depict a nidus comprising central sclerosis with circumferential radiolucency (arrowhead) at the olecranon/coronoid fossa of the humerus.

**Figure 3:**
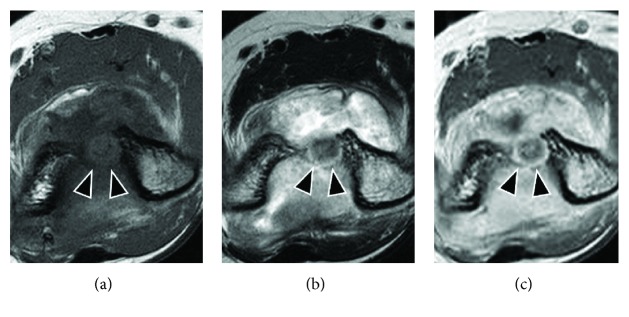
Axial T1-weighted (a), T2-weighted (b), and gadolinium-enhanced (c) images on preoperative magnetic resonance imaging. The round lesion shows slightly hyperintense signals compared to adjacent muscles on T1-weighted imaging and hyperintense to intermediate-intensity signals on T2-weighted sequences, with heterogeneous enhancement (arrowhead).

A 17-year-old right-handed female presented in our outpatient clinic with a 12-month history of pain and limited motion of her left elbow. The symptoms included mild pain during the day time that became increasingly severe at night. A physical examination revealed elbow contracture with a maximal extension of -20° and flexion of 125° (Figures 1(a)–1(d)). The patient failed conservative treatment performed at several clinics and was suspected of having monoarticular arthritis or tendinopathy. Plain radiograph and CT images taken at our clinic revealed a nidus at the olecranon/coronoid fossa of the distal humerus (Figures 2(a)–2(c)). The lesion had a maximal diameter of 9 mm, with central sclerosis of 6 mm. Magnetic resonance imaging was then performed, showing that the central lesion had slightly hyperintense signals compared to adjacent muscle on T1-weighted sequences and hyperintense to intermediate-intensity signals on T2-weighted sequences, with heterogeneous enhancement (Figures 3(a)–3(c)). In addition, proliferated synovial tissues throughout the joint were identified. Technetium-99m bone scintigraphy revealed a hot spot at the olecranon/coronoid fossa, corresponding to the lesion.

**Figure 4:**
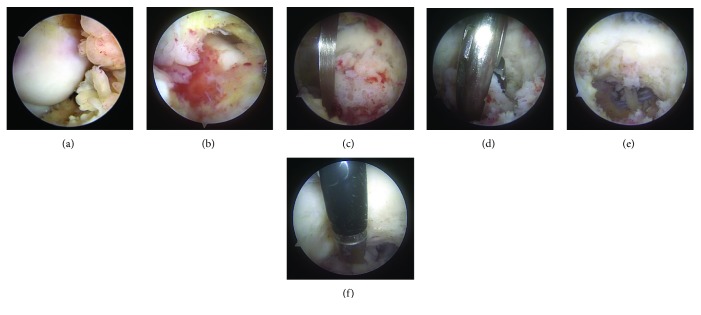
Arthroscopic findings. Proliferated synovial tissues were shown via anteromedial (a) and posterolateral (b) portals. The lesion was excised as an en bloc specimen (c, d, e). The margin was carefully treated to eliminate all traces of the tumor (f).

**Figure 5:**
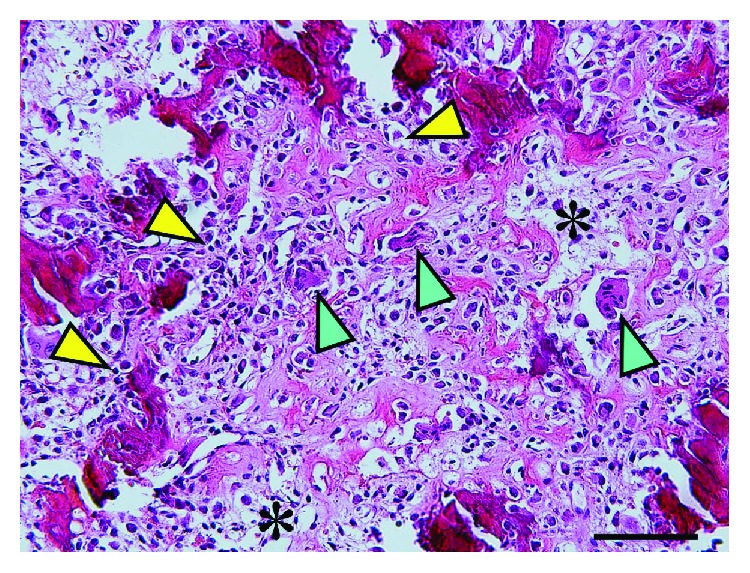
Histologic findings. Incompletely mineralized trabeculae rimmed by scattered osteoclasts (blue arrowhead) and irregularly shaped osteoblasts (yellow arrowhead). The intervening stroma (asterisk) is loose and fibrovascular (Hematoxylin-Eosin stain, bar represents 100 *μ*m).

**Figure 6:**
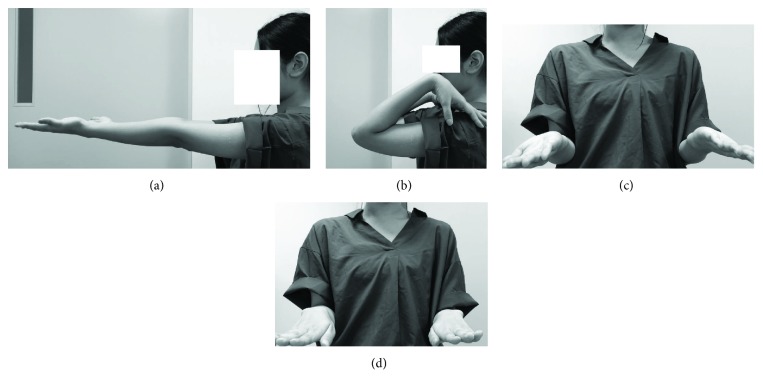
Ranges of motion at one-year follow-up. Elbow extension (a) and flexion (b). Forearm supination (c) and pronation (d).

Under a diagnosis of intra-articular OO at the elbow, the patient underwent arthroscopic treatment to excise the lesion. A routine anteromedial portal was created 1 cm anterior and 2 cm proximal to the medial epicondyle, and the scope was introduced. Severe proliferation of synovial tissues was observed throughout the joint space (Figure 4(a)). A 4 mm shaver blade was introduced through the anterolateral portal, which had been additionally created 1 cm anterior and directly lateral to the lateral epicondyle. Posterolateral and posteromedial portals were also created to treat the synovium located at the posterior capsule (Figure 4(b)). After thorough synovectomy, a tumorous lesion with a hyperemic surface was identified clearly through the anterior and posterior portals. The lesion was excised as an en bloc specimen using a bone chisel and cupped forceps (Figures 4(c)–4(e)). The margin was carefully debrided using the shaver and radiofrequency devices to eliminate all traces of the tumor (Figure 4(f)). No capsular release was performed. A histologic examination confirmed the diagnosis of OO (Figure 5). At the 12-month follow-up, the patient had no recurrence of elbow limitation or pain (Figures 6(a)–6(d)).

## 3. Discussion

We herein reported a case of arthroscopic excision for intra-articular OO of the elbow joint. The advantages of this technique include a low-invasive approach and easy accessibility to both the anterior and posterior aspects of the tumorous lesion. Under the classical open approach, in contrast, a relatively long incision is required, especially for determining the accurate degree of excision; consequently, this approach carries a risk of scar formation, residual pain, and contracture. Arthroscopically, thorough synovectomy enabled us to identify the tumorous lesion with hyperemic alteration. Arthroscopic maneuvers such as capsular release and debridement can be added to improve the joint motion. This patient did not undergo such release since the loss of the range of motion was considered mild. Bhatia [7] suggested anterior capsulotomy be performed to improve the access in cases with OOs at the radiocapitellar region or the proximal aspect of the distal humerus.

To our knowledge, 5 case reports and 1 case series have described a total of 17 patients who underwent arthroscopic treatment for elbow OOs [2, 6–10]; among them, only 5 patients had OO at the olecranon fossa. In the literature, arthroscopic treatment resulted in satisfactory outcomes in 14 patients, with 1 patient developing postoperative symptoms due to the insufficient removal of the lesion located at the anterior part of the trochlea [9]. As a knack in excising the whole lesion, Bhatia [7] mentioned the importance of adequate visualization and working access using a 70° view arthroscope and instruments with an angled tip to address the lesion.

The difficulty in diagnosing OO based on imaging findings has been mentioned in cases with severe inflammatory changes, such as a prominent periosteal reaction, exaggerated synovial hypertrophy, and joint effusion with extensive bone marrow and soft tissue edema [11]. In addition to the nidus identified on plain radiographs and CT images, additional assessments, including technetium-99m bone scintigraphy, can be useful for confirming OO [12]. However, the recognition of characteristic symptoms, including worsening pain at night and relief by nonsteroidal anti-inflammatory drugs, is crucial for clinicians to suspect OOs before performing any imaging assessments.

## 4. Conclusion

We herein report a 17-year-old female who underwent arthroscopic treatment for intra-articular OO located at the elbow. This case report describes the advantages of arthroscopic treatment, including a low-invasive approach and easy accessibility to the whole intra-articular space, which can provide clear visualization of the tumorous lesion.
